# Simplified immobilisation method for histidine-tagged enzymes in poly(methyl methacrylate) microfluidic devices

**DOI:** 10.1016/j.nbt.2017.12.004

**Published:** 2018-12-25

**Authors:** Gulsim Kulsharova, Nikolay Dimov, Marco P.C. Marques, Nicolas Szita, Frank Baganz

**Affiliations:** Department of Biochemical Engineering, University College London, London, WC1H 0AH, United Kingdom

**Keywords:** Microfluidics, Poly(methyl methacrylate) (PMMA), Enzyme immobilisation, Histidine-tagged enzyme, Transketolase

## Abstract

Poly(methyl methacrylate) (PMMA) microfluidic devices have become promising platforms for a wide range of applications. Here we report a simple method for immobilising histidine-tagged enzymes suitable for PMMA microfluidic devices. The 1-step-immobilisation described is based on the affinity of the His-tag/Ni-NTA interaction and does not require prior amination of the PMMA surface, unlike many existing protocols. We compared it with a 3-step immobilisation protocol involving amination of PMMA and linking NTA *via* a glutaraldehyde cross-linker. These methods were applied to immobilise transketolase (TK) in PMMA microfluidic devices. Binding efficiency studies showed that about 15% of the supplied TK was bound using the 1-step method and about 26% of the enzyme was bound by the 3-step method. However, the TK-catalysed reaction producing l-erythrulose performed in microfluidic devices showed that specific activity of TK in the device utilising the 1-step immobilisation method was approximately 30% higher than that of its counterpart. Reusability of the microfluidic device produced *via* the 1-step method was tested for three cycles of enzymatic reaction and at least 85% of the initial productivity was maintained. The device could be operated for up to 40 h in a continuous flow and on average 70% of the initial productivity was maintained. The simplified immobilisation method required fewer chemicals and less time for preparation of the immobilised microfluidic device compared to the 3-step method while achieving higher specific enzyme activity. The method represents a promising approach for the development of immobilised enzymatic microfluidic devices and could potentially be applied to combine protein purification with immobilisation.

## Highlights

• 1-step immobilisation for histidine-tagged enzymes onto PMMA surfaces developed.
• 1-step method achieved similar enzyme binding efficiency to established protocol.
• Model enzyme reaction gave higher specific activity and similar conversion yields.
• 1-step method requires less chemicals and time for PMMA surface preparation.
• 1-step method provides rapid and efficient way for enzyme immobilisation.

## Graphical abstract

**Figure:**
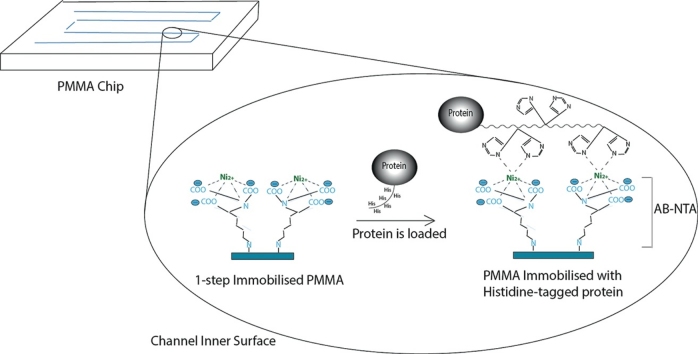

## Introduction

Microfluidic devices have found multiple applications in biotechnology, chemistry and chemical engineering [[1], [2], [3], [4], [5], [6]]. They offer many advantages such as large surface to volume ratio in microchannels, enhanced mass and heat transfer due to shorter diffusion paths [6], improved spatial and temporal reaction control [[7], [8]]. Their use has also increased in the context of enzymatic applications. Several techniques, either with free or immobilised enzymes, that demonstrate the potential of performing biocatalytic processes in microfluidic systems have been reported [[6], [7], [9], [10]]. For free enzymes in solution phase, there are some bottlenecks restricting their use, such as the need for an additional downstream unit operation to recover and reuse them, and long-term operational stability [7]. In contrast, immobilised enzymes offer advantages such as improved stability and reusability, without the need for purification of the catalyst from substrates and product [[11], [12], [13], [14]]. However, enzyme immobilisation on various surfaces can sometimes affect biocatalytic performance.

Various materials such as glass [[15], [16], [17]], polystyrene [18], silicon [[19], [20]], underivatized silica [[10], [21]] and poly(methyl methacrylate) (PMMA) [22], have already been used to immobilise enzymes in microfluidic devices. Of these, PMMA is very favorable for rapid prototyping and is widely used for fabrication of microfluidic devices due to its low price, biocompatibility, excellent optical transparency and attractive mechanical and chemical properties [23]. It also has potential in the creation of fully disposable microfluidic devices for a wide range of applications and is ideal for preparing “green microchips”, as it decomposes into reusable monomer at a high temperature [23]. Developing fast and simple immobilisation methods suitable for PMMA are of fundamental importance for the development of immobilised microfluidic systems.

A few approaches have been taken to immobilise enzymes in PMMA flow systems. Cerqueira et al. covalently immobilised glucose oxidase in PMMA microchannels using glutaraldehyde on a preliminarily aminated surface *via* polyethyleneimine [24]. Llopis et al. reported a covalent immobilisation method attaching active groups directly onto the PMMA surface of a microfluidic device using *N*-lithiodiaminoethane, by exposure to UV radiation preceded by *N*-(3-dimethylaminopropyl)-*N*-ethylcarbodiimide coupling of ethylenediamine addition [25]. Due to the inert nature of PMMA [26], the reported immobilisation methods were based on PMMA surface functionalisation with amine groups or required covalent binding of enzymes. The latter prevented reusability of the surface in contrast to reversible binding methods such as those based on His-tag/Ni-NTA interaction.

A His-tag directed immobilisation method applied to PMMA plug flow bioreactor was reported by Wollenberg et al. [27]. The authors used coupling of CYP2C9 to UV-activated PMMA *via* the Ni(II) chelator and His-tag on the enzyme for production of drug metabolites [27]. However, this method also contained surface functionalisation steps making the immobilisation in the PMMA microfluidic device more laborious and time consuming.

**Fig. 1:**
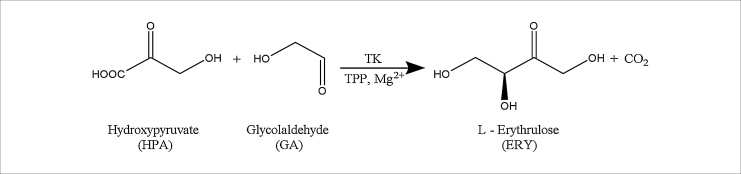
Reaction scheme of a transketolase (TK) catalysed reaction. Reaction scheme of the synthesis of l-erythrulose by TK using hydroxypyruvate and glycolaldehyde as substrates. The use of hydroxypyruvate as carbon donor enables the reaction to go to completion due to the release of carbon dioxide formed as by-product. The transketolase was immobilised on the surface of a PMMA device.

Here, we report a simplified procedure for His-tagged enzyme immobilisation on the surface of a PMMA microchannel using direct linking of *N*-(5-Amino-1-carboxypentyl) iminodiacetic acid (AB-NTA) molecules to a modified PMMA surface without the need for prior amination. Imaging and IR analysis of the surface were carried out to investigate the proposed simplified immobilisation method (here termed the 1-step immobilisation method). We compared the 1-step immobilisation with an established immobilisation protocol (here termed the 3-step immobilisation method) that requires an amination step and use of glutaraldehyde as a cross-linker prior to His-tag/Ni-NTA interaction. Both techniques were applied to immobilise His-tagged transketolase (TK) in a microfluidic device made of PMMA. TK was chosen due to its wide substrate tolerance and high enantio- and regio-specificity [28], which make it an attractive biocatalyst for the asymmetric synthesis of chiral metabolites [[29], [30], [31], [32], [33]] and highly relevant in the pharmaceutical industry. However, the method is applicable to a large variety of proteins that can be engineered with a His-tag [34]. The TK-catalysed conversion of hydroxypyruvate (HPA) and glycolaldehyde (GA) for production of the chiral ketoalcohol l-erythrulose (ERY) was chosen as a model reaction (Fig. 1). We compared specific activities of TK obtained from continuous reactions carried out in the microfluidic devices with TK immobilised using 1-step and 3-step immobilisation methods. Reusability and operational stability studies of the microfluidic device prepared by the 1-step immobilisation method were studied.

## Materials and methods

### Materials

(5*S*)-*N*-(5-Amino-1-carboxypentyl)iminodiacetic acid (AB-NTA) and glutaraldehyde were purchased from Insight Biotechnology (Wembley, UK). 1,6-Hexanediamine (HMDA) was obtained from Fisher Scientific (Leicestershire, UK). SDS 4–20% Tris-Glycine Mini-PROTEAN TGX™ Precast Gels and GFP recombinant *Aequorea victoria* protein were purchased from BioRad Laboratories Ltd. (Hertfordshire, UK) and Life Technologies (Paisley, UK), respectively. Poly(methyl methacrylate) (PMMA) was obtained from RS (Corby, UK) while BSA stock solution and the BCA protein assay kit were obtained from Thermo Fisher Scientific (UK). All other reagents were purchased from Sigma-Aldrich (UK).

### Microfluidic device fabrication

All components were designed using Adobe Illustrator CS6 software (Adobe Systems Inc., USA). The device was comprised of two rigid 2 mm PMMA layers. One of the layers has a channel with additional ridges for increasing the surface area to volume ratio and the total length is 280 mm (Fig. S1).The channels and cutouts were fabricated using a CO_2_ laser marking head (Zing, USA) and the layers were thermally bonded (2 h, 102.5 °C). Interconnect ports were laser cut from 6 mm thick PMMA, with two holes tapped with a M3 thread for attachment of the connector to the device, and an M6 threaded hole to allow standard connection tubing (P-221, Upchurch Scientific, Oak Harbor, USA) to be attached.

To determine the experimental volume of the microfluidic device, the device with clean and dry microchannel was connected to a syringe pump (AL 1000-220). Water was pumped at a fixed flow rate and time that it takes for the water to flow from the inlet to the outlet was measured. Reactor volume was calculated by multiplying the given flow rate by the measured time.

### Biocatalyst preparation

Transketolase stocks of *E. coli* BL21gold (DE3) with plasmid pQR791 (His_6_-TK) were produced in-house and stored at −80 °C in LB broth containing 50% (v/v) glycerol. In order to obtain higher enzyme yields, MagicMedia *E.coli* expression medium (ThermoFisher Scientific, UK) was used. The complete MagicMedia medium was prepared using the supplied kit components according to its manual. The protocol for biocatalyst preparation was similar to that previously described [35]. Briefly, overnight cultures of *E. coli* were prepared in flasks containing 10 ml of LB broth with 150 μg mL^−1^ ampicillin. Cells were sub-cultured using 1% (v/v) inoculum in 1 l baffled flasks containing 200 ml of the prepared complete MagicMedia medium with 150 μg mL^−1^ ampicillin and incubated at 37 °C and 250 rpm until the bacterial growth reached stationary phase. Cells were harvested by centrifugation at 4000 rpm for 20 min. The cell pellets were resuspended in 50 mM Tris-HCl pH 7.0 and sonicated on ice (Soniprep 150, MSE Sanyo, Japan). The suspension was centrifuged at 13,000 rpm and 4 °C for 20 min. The supernatant was filtered using PVDF syringe filters (Whatman, Maidstone, UK) with a molecular weight cutoff of 0.2 μm and stored at −20 °C until further use.

### Preparation of poly(methyl methacrylate) microfluidic device with immobilised his-tagged transketolase

#### 3 -step immobilisation protocol

A PMMA microfluidic device channel was functionalised and immobilised with His-tagged enzyme according to protocols adapted from Fixe et al. and Oshige et al. [[34], [36]]. Briefly, a channel in the PMMA microfluidic device was flushed and filled with isopropanol (99%) and incubated at 30 °C for 3 h. The microfluidic device channel was then rinsed thoroughly with Milli-Q water and incubated with 10% (v/v) hexamethylene-diamine (HMDA) in 100 mM borate buffer pH 11.5, for 2 h. The channel was then thoroughly flushed with Milli-Q water for several channel volumes. Afterwards, the channel was incubated with 1% (v/v) glutaraldehyde overnight at 37 °C. Another overnight incubation at 37 °C was done with a 0.05% (w/v) solution of *N*-(5-amino-1-carboxy-pentyl) iminodiacetic acid (AB-NTA) in 0.1 M HEPES buffer, pH 8.0. Then, the channel was flushed with Milli-Q water using syringe pump AL 1000-220 (World Precision Instruments, USA) at flow rates of 20 μl min^−1^. Finally, a solution of 0.5 M NiCl_2_ was pumped through the microchannel at a flow rate of 10 μl min^−1^ for 1 h followed by a Milli-Q wash.

#### 1 -step immobilisation protocol

1-step immobilisation protocol was adapted from the 3-step immobilisation by direct conjugation of AB-NTA molecules to available methyl ester bonds on PMMA surface. For this purpose, the channel was filled with isopropanol (99%) and incubated at 30 °C for 3 h. After rinsing with Milli-Q water, the channel was incubated overnight with 0.05% (w/v) AB-NTA in 0.1 M HEPES buffer, pH 8.0. Analogously to the 3-step immobilisation, the channel was rinsed with Milli-Q water and then a 0.5 M NiCl_2_ solution was pumped through the microchannel at a flow rate of 10 μl min^−1^ for 1 h. The channel was washed with Milli-Q water before TK immobilisation.

### Transketolase immobilisation and elution

Cell lysates containing TK ranging from 20% to 28% (w/w) were loaded at a protein concentration of 14.8 ± 2.6 mg ml^−1^ and 14.3 ± 3.1 mg ml^−1^ for the 1-step and 3-step immobilised microfluidic devices, respectively. The lysates were pumped into the microchannels using a syringe pump AL 1000-220 (World Precision Instruments, USA) at a flow rate of 5 μl min^−1^ at 4 °C for several channel volumes. After 1 h a solution of 50 mM Tris-HCl, pH 7.5 was pumped through the microchannels at 20 μl min^−1^ to remove non-specifically bound enzyme. Samples were collected periodically and assayed for protein content. After operational stability studies, the bound enzyme was removed by treating the channel with EDTA elution buffer (50 mM EDTA; pH 8.0) at 20 μl min^−1^ for at least 2 channel volumes. Collected samples were concentrated down to 75 μl volume using Amicon Ultra Centrifugal filters (30,000 NMWL).

### Characterisation of immobilisation surface

For Scanning Electron Microscopy (SEM) imaging studies, three PMMA devices were fabricated following the procedure described earlier. One microchannel surface was used as a control and remained untreated. The second and third microfluidic device channel surfaces were modified using the 1-step immobilisation protocol and washed to remove any excess or non-specific binding. After this, the third microfluidic channel surface was used for TK immobilisation as described previously and washed with Milli-Q water to remove any non-specifically bound enzyme. The channel surfaces were dried and sputter-coated by a mix of gold and platinum nanoparticle layer to allow good conductivity on the surface. SEM imaging of the pre-treated samples was carried out using JEOL 5610LV system with magnifications ranging from 100 to 20 000-fold.

Fourier transform infrared (FT-IR) spectra of bare PMMA and the AB-NTA conjugated PMMA surfaces were recorded on Bruker platinum ATR. Samples were two microchannel surfaces that were laser cut into 2 mm PMMA slabs following the fabrication procedure described earlier. The bare PMMA surface was cleaned and dried before measurement. Following the 1-step immobilisation procedure on a bare PMMA surface and after incubation with AB-NTA, the surface was washed with Milli-Q water to remove any non-bound AB-NTA molecules. The surface was dried before FT-IR spectroscopy.

### Transketolase reactions in microfluidic device

Transketolase reaction in the microfluidic device was carried out at 4 °C. A cofactor solution of 4.8 mM thiamine diphosphate (ThDP) and 19.6 mM magnesium dichloride (MgCl_2_) in 50 mM Tris-HCL, pH 7.0 was pumped through the microchannel at 10 μl min^−1^ (KDS100, KD Scientific, Holliston, USA) for 30 min. Afterwards, a substrate mix of 12.5 mM glycolaldehyde (GA) and 12.5 mM hydroxypyruvate (HPA) in 50 mM Tris-HCL pH 7.0 was flown through the reactor at flow rates ranging from 2.3 to 30 μl min^−1^ (mean residence times of 25 to 1 min, respectively). Samples generated from each flow rate were collected into Eppendorf tubes containing 0.1% (v/v) trifluoroacetic acid and analysed by HPLC.

### Reusability and operational enzyme stability studies

After each reaction run, the microfluidic device was washed with 50 mM EDTA pH 8.0 at 20 μl min^−1^ for two microchannel volumes at room temperature. Samples were collected and assayed for protein content. The microfluidic device was stored at −20 °C until further use. For the next cycle of immobilisation, the microfluidic device was first washed with Milli-Q water at 20 μl min^−1^ and then the surface was regenerated with NiCl_2_ as described in 1-step immobilisation method section. Loading of TK was carried out as mentioned in transketolase immobilisation and elution section.

Operational stability study was conducted in a microfluidic device with TK immobilised through 1-step immobilisation. After TK immobilisation and washing steps, TK reaction was carried out under a constant flow rate of 5 μl min^−1^ for up to 40 h. Samples were collected at various times into tubes with 0.1% (v/v) trifluoroacetic acid and analysed for ERY production by HPLC.

### Analytics

#### SDS-PAGE and protein concentration determination

SDS-PAGE protein analysis was performed using a Mini-155 Protean II system (Bio-Rad Laboratories Inc., Hemel Hempstead, UK) with SDS 12% Tris-Glycine (BioRad, UK) pre-cast gels using 10x Tris/Glycine/SDS electrophoresis buffer (BioRad, UK). Gels were stained with Instant Blue (Expedeon Ltd., Cambridge, UK). Destaining was performed in Milli-Q water overnight and a *Gel-Doc-it* bioimaging system (Bioimaging systems, Cambridge) was used to visualize the gels.

Protein concentration was determined in triplicates using the Micro BCA protein assay kit (Thermo Scientific, UK) according to the manufacturer instructions. Absorbance measurements at 561 nm were carried out on an ATI Unicam UV/VIS spectrophotometer (Spectronic, Leeds, UK). Protein concentration was quantified using a calibration curve with bovine serum albumin as standard.

#### TK mass and specific activities of transketolase

The amount of eluted TK was determined by SDS-PAGE densitometry. Densitometry of samples electrophoresed on a 12% SDS-PAGE gel (Bio-Rad) was used. To calculate the mass of eluted enzyme in elution studies, imaging and quantification was done using GE Amersham Imager 600. A range of concentrations of commercial BSA was run on an SDS-Page gel to obtain a standard curve based on integrated band density for calibration of the enzyme concentration. BSA concentration of 0.5 mg mL^−1^ was used as a reference band on all other gels to account for variation in density obtained from different gels.

For enzyme binding efficiency and consequent specific activity determination studies previously imaged gels were analysed using ImageQuant TL Version 8.1 to calculate the TK band density relative to the total band density. Total protein in the samples was determined using a BCA assay.

### Substrate and product quantification of TK-catalysed reactions

l-erythrulose (l-ERY) and hydroxypyruvate (HPA) were quantified with HPLC (Ultimate 3000, Thermo, UK) using an Aminex HPX-87 column (300 mm × 7.8 mm, Bio-Rad, UK) at 60 °C using an isocratic flow of 0.1% (v/v) aqueous trifluoroacetic acid at 0.6 mL min^−1^. Compounds were detected at 210 nm.

## Results and discussion

### Immobilisation of transketolase on to poly(methyl methacrylate) surface

Many protocols for immobilising enzymes on to polymeric materials require prior surface modification, since the material surface does not contain suitable functional groups in their native form [37]. For example, the protocol for immobilisation of His-tagged proteins on various polymers developed by Oshige et al. requires surface amination in order to bind glutaraldehyde that is used as a cross-linker enabling enzyme binding *via* Ni-NTA [34].

**Fig. 2:**
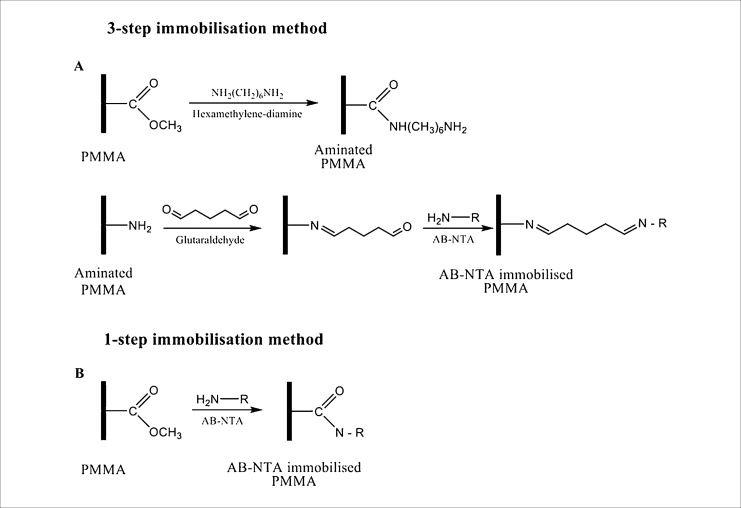
1-step and 3-step immobilisation chemistries on PMMA surface. (A) 3-step immobilisation chemistry. The first line corresponds to amination chemistry of the surface. The available methyl esters of PMMA, under basic pH conditions, react with an electron donor (N) present on the hexamethylene-diamine (HMDA), producing primary amines on the surface. The second line of the scheme represents the linking step of primary amine bonds formed on PMMA surface with amine bonds of AB-NTA molecule using the cross-linker glutaraldehyde. (B) 1-step immobilisation chemistry. AB-NTA molecule substitutes the HMDA step corresponding to 3-step immobilisation procedure, and amine bonds of AB-NTA molecule react with the available methyl esters on the PMMA surface, under basic pH conditions. These 1-step and 3-step immobilisation procedures produce a functionalised PMMA surface that subsequently is used for immobilising histidine-tagged enzymes.

Here, a 3-step immobilisation method adapted from Oshige et al. based on amination of PMMA was investigated first [34]. The protocol included amination of the PMMA surface using hexamethylene-diamine (HMDA). Glutaraldehyde was used as a cross-linker to conjugate amine bonds of *N*-(5-Amino-1-carboxy-pentyl) iminodiacetic acid (AB-NTA) to the aminated PMMA surface as shown in Fig. 2A. However, application of this method was time-consuming, taking two overnight incubations to complete the surface treatment.

To establish a simpler protocol with concomitant reduction of surface modification steps and the time involved, we developed an alternative method where AB-NTA binds to available methyl esters of PMMA under basic pH conditions forming amide bonds as shown in Fig. 2B. Conjugating AB-NTA onto the surface of PMMA eliminates the amination step required in existing protocols for enzyme immobilisation on PMMA and the need for glutaraldehyde required in the 3-step immobilisation method.

### Characterisation of PMMA microchannel surfaces using scanning electron microscopy and infrared spectroscopy

**Fig. 3:**
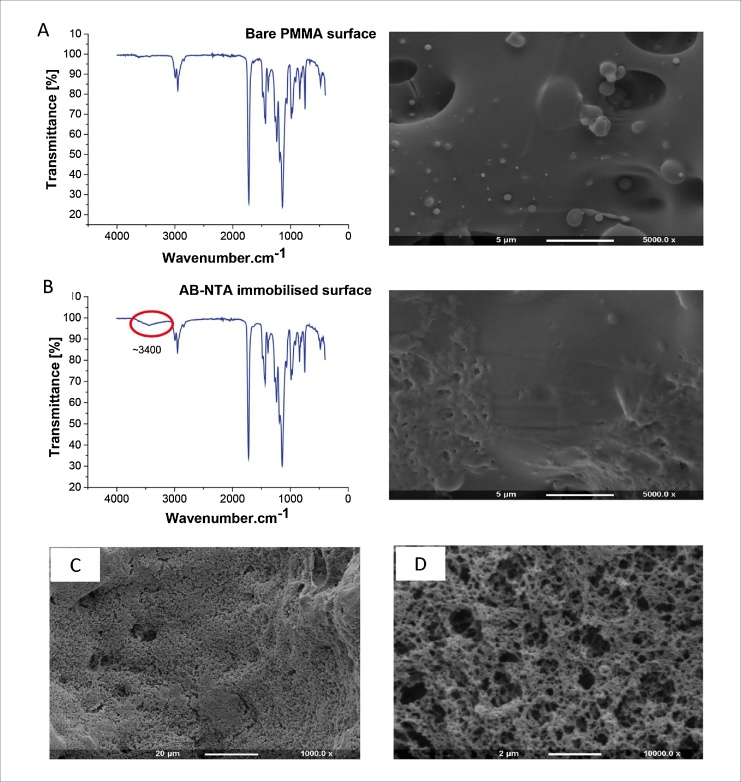
Set of Fourier transform infrared spectroscopy spectra and respective scanning electron microscopy (SEM) images of (A) bare PMMA microchannel surface; (B) microchannel surface treated with AB-NTA according to 1-step immobilisation method (C) 1000× and (D) 10,000× magnifications of PMMA channel surface after immobilisation of transketolase using 1-step immobilisation method.

To confirm the presence of AB-NTA and consequent immobilisation of TK using the 1-step immobilisation method, modified PMMA surfaces were investigated using infrared spectroscopy and scanning electron microscopy. Fig. 3 shows Fourier transform infrared (FT-IR) spectra and respective scanning electron microscopy (SEM) images of unmodified PMMA channel surface (A), AB-NTA treated channel surface (B), and of modified PMMA surface with TK immobilised *via* the 1-step immobilisation method (without IR spectra) (C, D).

The surface of the open microchannel before modification steps showed roughness and re-settled material particles and small cavities due to the laser cutting microfabrication process (Fig. 3A SEM image). Roughness is very favorable for our application, as it potentially increases the effective area to be modified. The FT-IR spectrum of unmodified PMMA channel shown in Fig. 3A presents a set of bands that are characteristic of pure PMMA surfaces [38].

The coupling of AB-NTA to the PMMA surface under basic pH conditions was confirmed by the FT-IR spectrum (Fig. 3B), where the peak at ∼3400 cm^−1^ can be assigned to the N—H stretching vibrations due to amide bond formation [36]. As can be seen in the corresponding SEM image of the channel, the presence of AB-NTA on inner walls did not lead to significant changes in surface profile (Fig. 3B SEM image).

Fig. 3(C) and (D) shows the SEM images of surfaces with TK immobilised *via* the 1-step method on to the PMMA microchannel surface. These images showed a porous structure that was distinctly different from the AB-NTA treated surface (B) suggesting that these were enzyme structures bound on to the surface. We assume that, due to the apparent porous surface of the microchannel, the attached enzyme formed an uneven layer or multiple layers.

### Enzyme binding efficiency in immobilised microfluidic devices

A microfluidic device fabricated out of PMMA with a microchannel and additional grooves for the enhanced surface area was used. The internal surface area of the microfluidic channel was calculated as 1040 mm^2^ (Supplementary materials, Section S1). The calculated volume was 80 μl assuming sharp edges of the designed grooves inside the microchannel. However, the experimentally determined volume was only 56 μl. The difference is likely to be due to the non-rectangular profiles of the microchannel surface, which can be seen from Fig. S2 and therefore the experimentally determined volume was used in all calculations in this work.

**Fig. 4:**
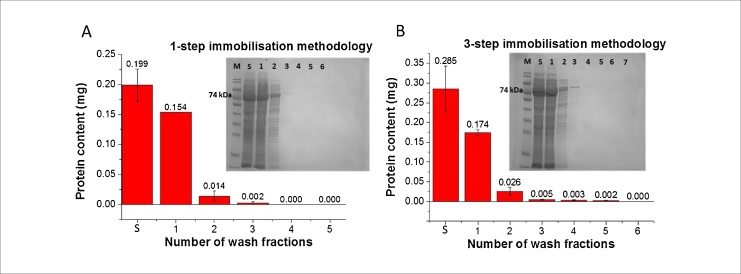
Quantification of transketolase (TK) protein concentration to determine enzyme binding efficiency of immobilised microfluidic devices prepared by 1-step and 3-step immobilisation methods. Protein amounts loaded into the microfluidic device and collected in flow-through (wash) fractions from the microchannels with immobilised TK via (A) 1-step and (B) 3-step methods. Inset: SDS-PAGE gel of protein samples. Lane M corresponds to SDS marker with the 74 kDa band indicative of the size of the TK monomer (72.5 kDa), Lane S shows the amount of protein loaded (S = ∼0.20 ± 0.03 mg TK for 1-step and 0.29 ± 0.06 mg TK for 3-step immobilised microfluidic devices), and Lanes 1–7 represent the protein content in collected wash fractions. Final immobilised amount of TK enzyme in microfluidic devices using 1-step and 3-step methods was calculated to be 29 ± 15 μg and 75 ± 36 μg, respectively. Protein detection was done in triplicates. Error bars represent standard deviation above the mean (n = 3).

Immobilisation of TK was carried out using a cell lysate obtained from an *E. coli* shake flask culture overexpressing a wild-type TK enzyme. Clarified cell lysates at concentrations of 14.76 and 14.31 mg ml^−1^ (containing 20% and 28% TK respectively determined *via* densitometry) were used for loading into the microfluidic devices modified by 1-step- or 3-step immobilisation methods respectively. The amount of enzyme retained in the microchannel was determined by a mass balance between the amount of enzyme loaded at the inlet and that measured at the outlet and in the wash fractions. In total, 6 to 7 washing steps were performed with each collected sample containing a solution of one channel volume with the outlet tubing volume (112 μl for 1-step and 124 μl for 3-step immobilised microfluidic devices, respectively). Protein quantification of wash fractions after enzyme immobilisation is shown in Fig. 4A and B. Using SDS-PAGE densitometric analysis, the percentages of TK relative to total protein in each lane of SDS-PAGE gels depicted in the insets of Fig. 4A and B were determined and used to calculate TK content in each wash fraction. No protein was detected after the third wash of the microchannel immobilised with TK *via* the 1-step immobilisation method (Fig. 4A inset), while the 3-step microfluidic device samples showed that small amounts of protein were still detected up to the fifth wash fraction (Fig. 4B inset). Note that a control study of TK loading into a bare, non-modified PMMA microfluidic device showed protein wash outs for the entire 7 washes and no retention of the enzyme in the channel was observed, as expected (Fig. S3). Based on the mass balance, the amount of bound TK in the microchannels was estimated to be 29 ± 15 μg for 1-step and 75 ± 37 μg for 3-step immobilised TK microfluidic devices, respectively. These amounts represented an average binding efficiency of ∼15% for the 1-step and ∼26% for the 3-step methods and suggested that approximately 6 and 15 times more enzyme was immobilised in the microfluidic device, respectively, than the theoretically estimated amount for a microchannel of such geometry. The theoretical amount was estimated assuming monolayer immobilisation of TK in the microchannel and microfluidic device surface was found to accommodate approximately 5 μg of TK. Details of calculations are shown in the Supplementary materials, Section S1. The finding supports our interpretation of SEM images that the porous structure formed increases the available area for enzyme binding significantly. It is also in agreement with Miyazaki et al. [39] who reported 8–10 fold higher enzyme amount relative to monolayer coverage for a His-tagged l-lactic dehydrogenase immobilised in a derivatised silica microchannel. Additionally, elution of TK immobilised in the microchannel using the 1-step immobilisation method produced a faint band corresponding to the TK molecular weight (Fig. S4). Densitometric analysis of the band showed that approximately 9 μg of TK was recovered from the elution of the microfluidic device prepared using the 1-step method, which comprised ∼31% of the average amount of TK calculated from the mass balance. This eluted amount of TK was almost twice that of the theoretical value calculated from the geometry of the channel. The difference between the bound TK calculated from the mass balance and that obtained from the eluted band is probably due to the difficulty of accurately quantifying such small amounts of enzyme.

### Transketolase reactions in continuous modes

In order to compare specific activities of TK in two PMMA microfluidic devices immobilised through the 1-step and 3-step methods, l-erythrulose production was investigated. Previously, the TK-catalysed reaction was characterised in continuous flow microreactors with the enzyme immobilised either on the microchannel walls or on Ni-NTA agarose beads [[10], [21]]. In contrast, here a different immobilisation method suitable for PMMA was used and the device was operated at higher flow rates ranging from 2 to ∼30 μl min^−1^. In addition, to ensure the prolonged stability of the enzyme over extended periods of time the continuous flow reaction was run at 4 °C instead of room temperaturein order to minimise possible thermal deactivation of the enzyme.

**Fig. 5:**
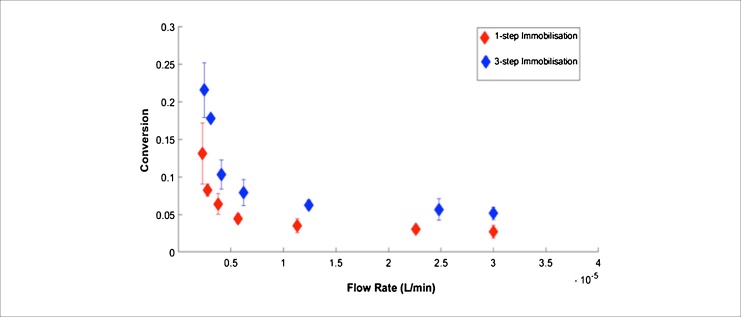
Conversion of glycolaldehyde (GA) and hydroxypyruvate (HPA) to l-erythrulose (l-ERY) at 4 °C as a function of flow rate in the PMMA microfluidic devices. Comparison of product formation obtained from microfluidic devices with transketolase (TK) immobilsed via 1-step and 3-step immobilisation techniques. The specific activity values derived from this data were 124 ± 13 and 88 ± 11 nmol min^−1^ mg^−1^ for 1-step and 3-step immobilisation, respectively. Error bars represent one standard deviation above the mean (n = 2). TK-catalysed reactions were performed using equimolar concentrations of GA and HPA (12.5 mM) each.

Dependence of enzyme conversion on flow rate shown in Fig. 5 which yielded similar profiles for microfluidic devices immobilised with 1-step or 3-step methods. Specific activities of TK in the two devices were found to be 124 ± 13 and 88 ± 11 nmol mg^−1^ min^−1^ for 1-step and 3-step immobilisation, respectively. Data were derived from the product formation determined as a function of residence time shown in Fig. S5 in Supplementary materials. For immobilised TK on Ni-NTA agarose beads, Halim et al. [21] previously reported a specific activity of 8.20 μmol mg^−1^ min^−1^, while Matosevic et al. [10] reported a specific enzyme activity of 0.8 μmol mg^−1^ min^−1^ for TK immobilised on the walls of a silica capillary. The reduced specific activity found in this study can be explained by the lower temperature (4 °C) used in comparison with previous studies that were carried out at room temperature.

Furthermore, the immobilised TK retained only ∼4% of the specific activity of the free enzyme in solution, found to be 3.38 μmol mg^−1^ min^−1^ (Fig. S6). This is in line with earlier data of Matosevic [40], who also reported that 4% of the free enzyme activity was retained using His-tagged immobilisation. For covalent attachment *via* cross-linking with glutarldehyde onto the amino-silanized surface Thomsen et al. reported that immobilised β-glycoside hydrolase CelB in a microreactor retained only about 3% of the specific activity of the free enzyme, while the CelB attached to silanized macroporous glass beads in batch, which employed the same immobilisation technique, retained 35% of the free enzyme specific activity [41]. Such large differences between immobilised and free enzyme specific activities could be caused by mass transfer limitations due to an unfavorable conformation of the immobilised enzyme and/or multi-layer attachment and possibly some inactivation of the enzyme in an immobilised form [38].

### Reusability of immobilised microfluidic device prepared by 1-step method

To examine reusability of the 1-step immobilisation layer, the performance of the microfluidic device was tested over multiple immobilisation-elution-regeneration cycles. Previously reported immobilised microreactors have shown better retention of enzyme activity than using enzymes in free solutions. For instance, Thomsen et al. demonstrated consistent productivity of a β-glycoside hydrolase CelB-immobilised microreactor over 5 days [38] and. Matosevic et al. showed successful reusability of a silica-based immobilised microreactor over 5 cycles [10]. Here, the suitability of 1-step immobilisation protocol for multiple usages over longer periods of time was investigated. The reusability of the microfluidic device with TK immobilised *via* 1-step method was studied by using the same microchannel after eluting the enzyme and reusing the microreactor in three successive runs each with fresh enzyme. Productivity levels close to 100% were maintained over 3 cycles operated at a flow rate of 10 μl min^−1^, although a reduction to 85% was observed in the second reuse (Fig. S7A). It can be concluded that the immobilisation surface was stable and that the activity of TK was maintained for at least 21 days.

In addition, the operational stability of the TK-immobilised microfluidic device *via* 1-step immobilisation method was investigated under continuous flow over 40 h at a flow rate of 5 μl min^−1^. Samples were taken after 1, 14 and 39 h to measure the conversion of HPA and GA to ERY product. The productivity of the TK immobilised in the device decreased to around 65% after 14 h but on average remained around 70% for the duration of the study (Fig. S7B). The results are similar to previously obtained values of operational stability of TK immobilised in a packed-bed reactor, where productivity dropped to 76% after 48 h of reaction time [19]. However, it is worth noting that flow rates used in this study were 5 times higher than those in the reported study (1 μl min^−1^), which may explain the slight difference in productivities of the immobilised TK.

## Conclusions

In this report, 1-step immobilisation of a His-tagged enzyme was investigated as a fast and simple alternative to existing methods for immobilising enzymes in PMMA microfluidic devices. The 1-step method was compared to the more common 3-step immobilisation of His-tagged enzymes in PMMA microchannels. We assessed the chemistries of the two immobilisation techniques and corresponding surface preparation steps, their enzyme binding efficiencies in microfluidic devices as well as the specific activities of the immobilised enzyme and conversion yields (Table 1). The 1-step immobilisation method holds potential advantages over other protocols. One of its advantages is the much shorter preparation time. In addition, the number of chemicals required for 1-step immobilisation is significantly less, since this method does not require separate surface amination of PMMA, unlike the 3-step immobilisation protocol. As a result, the immobilisation cost may potentially be lower. Furthermore, the presented method requires fewer wash steps. The simplified 1-step protocol yielded approximately 10% lower enzyme binding efficiency than the 3-step immobilisation method, but produced similar l-erythrulose conversions as a function of flow rate due to higher specific enzyme activity. The 1-step method presents a viable approach for the development of enzymatic microfluidic devices and could potentially be applied to combine enzyme purification with immobilisation of His-tagged proteins from crude cell extracts. Potentially, our novel 1-step immobilisation protocol could be applied for antibody purification and also fabrication of antibody arrays towards the development of cost efficient immunosorbent assays.

**Table 1:** Comparison of key characteristics of the 1-step and 3-step immobilisation of His-tagged transketolase in poly(methyl methacrylate) PMMA microfluidic devices.

Characteristic	1-step immobilisation	3-step immobilisation
Preparation time	1 day	2–3 days
Number of chemicals required	3	7
Binding efficiency, (%)	∼15	∼26
Specific activity, (nmol mg^−1^ min^−1^)	124 ± 13	88 ± 11

## Conflicts of interest

The authors declare no conflict of interest.

## Author contributions

G.K. and F. B. conceived, designed and G.K. performed the experiments; G.K. N.D., M.M. and F.B. analysed the data; G.K. wrote the manuscript with input from F.B., N.D. and M.M.; N.S and F.B. edited the paper.
