# Supplementary material for: Simplified immobilisation method for histidine-tagged enzymes in poly(methyl methacrylate) microfluidic devices
Source: N Biotechnol. 2018 Dec 25;47:31–8. doi: 10.1016/j.nbt.2017.12.004 (PMC6191535; doi:10.1016/j.nbt.2017.12.004)
Supplement: Supplementary file 1 [file mmc1.docx]

## Supplementary Materials

###
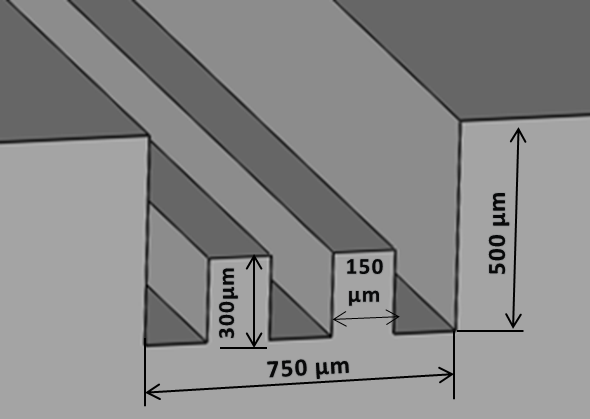
Section S1: Calculation of internal surface area of the microfluidic device and minimum amount of bound enzyme on the internal surface of the channel

Figure S1. Cross-section of the microfluidic device channel. Poly(methyl methacrylate) (PMMA) microfluidic device has a channel with additional ridges for increasing the surface area to volume ratio. The total length of the microchannel is 280 mm.

A completely flat microchannel surface was assumed. The total internal surface area of the microchannel was calculated as follows. The cross section of the microchannel (Figure S1) was regarded as a rectangle with additional rectangular grooves. By using designed length of the microchannel (280 mm), perimeters of rectangles were calculated representing surface areas of groove and inner microchannel walls as follows:

$$S_{top}= S_{bottom}=280 mm \text{×} 0.75 mm=210 {mm}^{2}$$

$$S_{side}=280 mm \text{×} 0.5 mm=140 {mm}^{2}$$

$$S_{groove side surface}=280 mm \text{×} 0.3 mm=84 {mm}^{2}$$

$$S_{total}=S_{top}+ S_{bottom}+{2 \times S}_{side}+{4 \times S}_{groove side surface}=1036 {mm}^{2}$$

Additional surface area is provided by grooves, and we account for the surface area of the sides of grooves only, as the top surface of groove is considered in *S_bottom_*. Thus, the total surface area was estimated as 1036 mm^2^.

Assuming 1-step and 3-step immobilisation methods give a monolayer of TK, and the surface accommodates only bonds that are perpendicular to the surface of the channel, the amount of TK immobilised was estimated. This was the theoretical amount based on a model of monolayer enzyme coverage when bound to a plain surface using the molecular weight of the dimeric (active) form of transketolase (MW = 145 kDa). Assuming saturated binding of His-tagged TK on the inner surface of microfluidic device channel forms a monolayer of spherical molecules, lengths of bonds being the same; the quantity of bound enzyme was estimated. The total internal surface area of the microchannel is 1036 mm^2^. Fischer *et al*. [1] reported that the average density for proteins with MW more than 20 kDa is approximately 1.4 g∙cm^-3^. The radius of each TK molecule then can be estimated as follows.

$$\boldsymbol{Protein volume\approx}\frac{\boldsymbol{4}}{\boldsymbol{3}}\boldsymbol{\pi r}^{\boldsymbol{3}}$$

or,

$$\frac{\boldsymbol{MW}}{\boldsymbol{\rho}\boldsymbol{N}_{\boldsymbol{a}}}\boldsymbol{=}\frac{\boldsymbol{4}}{\boldsymbol{3}}\boldsymbol{\pi}\boldsymbol{r}^{\boldsymbol{3}}$$

By using

$$\boldsymbol{r=}\sqrt[\boldsymbol{3}]{\frac{\boldsymbol{3}}{\boldsymbol{4 \pi}}\frac{\boldsymbol{MW}}{\boldsymbol{\rho}\boldsymbol{N}_{\boldsymbol{a}}}}\boldsymbol{=}\sqrt[\boldsymbol{3}]{\frac{\boldsymbol{3}}{\boldsymbol{4 \pi}}\frac{\boldsymbol{72,500}}{\boldsymbol{1.4\times}\left( \boldsymbol{6\times}\boldsymbol{10}^{\boldsymbol{23}} \right)}}\boldsymbol{=0.0027 \mu m}$$

Finding the protein area by $\boldsymbol{A=}\boldsymbol{\pi r}^{\boldsymbol{2}}$ and knowing the total internal surface area of the microchannel (stated above), we can estimate how many protein molecules can be accommodated by the internal surface.

$$\boldsymbol{N=}\frac{\boldsymbol{1036}\boldsymbol{\times10}^{\boldsymbol{6}}\boldsymbol{\mu m}^{\boldsymbol{2}}}{\boldsymbol{2.36}\boldsymbol{\times10}^{\boldsymbol{-5}} \boldsymbol{\mu m}^{\boldsymbol{2}}}\boldsymbol{= 4.4 \times}\boldsymbol{10}^{\boldsymbol{13}}\boldsymbol{molecules}$$

$$\boldsymbol{Moles of TK=}\frac{\boldsymbol{Molecules}}{\boldsymbol{N}_{\boldsymbol{a}}}\boldsymbol{=}\frac{\boldsymbol{4.4 \times}\boldsymbol{10}^{\boldsymbol{13}}}{\boldsymbol{6.02\times}\boldsymbol{10}^{\boldsymbol{23}}}\boldsymbol{=0.73 \times}\boldsymbol{10}^{\boldsymbol{-10}}\boldsymbol{mol}$$

Thus, the mass of TK immobilised in the microchannel was estimated as:

$\boldsymbol{Mass of TK=Moles \times MW=0.73 \times}\boldsymbol{10}^{\boldsymbol{-10}}\boldsymbol{\times72,500= 52925 \times}\boldsymbol{10}^{\boldsymbol{-10}}$ = 5.3 μg

Thus, theoretical amount of enzyme, which can be bound under the assumption that TK molecules form a monolayer on the inner surface of a channel, which is assumed to be even and flat, is 5.3 μg.


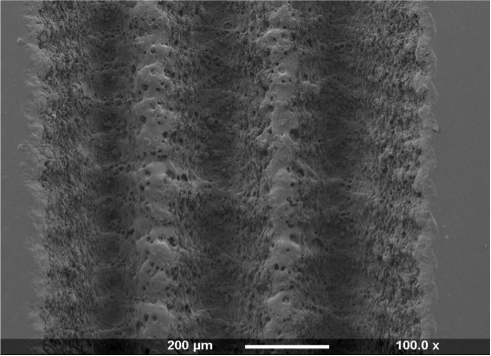


Figure S2. Scanning electron microscopy (SEM) image of the inner surface of an untreated PMMA channel. 100x magnification shows the full width of the channel section. The figure suggests that the cross-section of the channel is in fact approximately triangular, instead of expected rectangular shape.


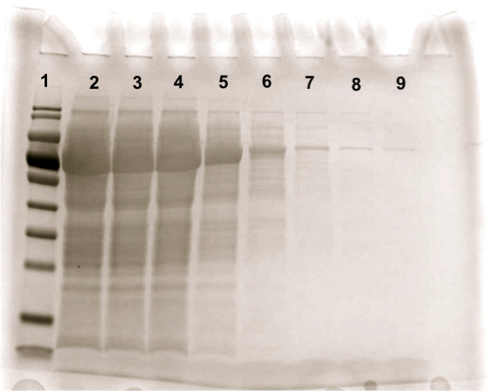


Figure S3. SDS-PAGE gel of collected flow through fractions from the control microfluidic device with no surface treatment of the microchannel. SDS-PAGE gel of samples of loaded TK lysate and the flow through fractions. Lane 1: SDS Marker; Lane 2, 3: 2x diluted TK lysate sample and 1st wash out fraction, respectively; Lane 4-9: wash fractions. Protein wash out is observed in the all wash out fractions indicating no protein retention in the microfluidic device channel as expected.


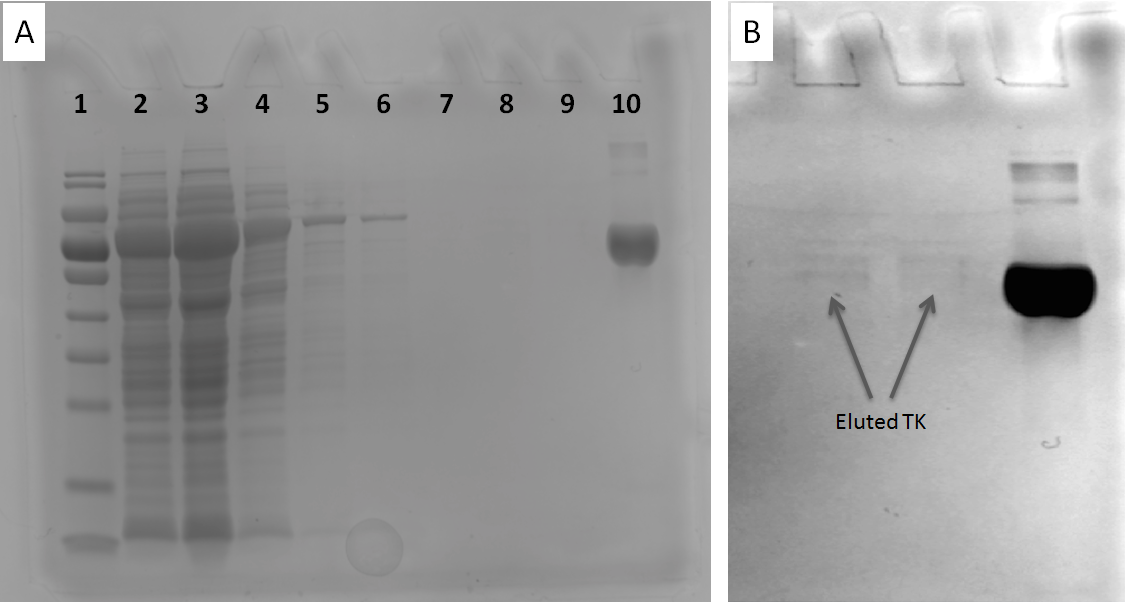


Figure S4. SDS-PAGE gel of collected flow through fractions from the microchannels with immobilised TK *via* 1-step immobilisation method and of elution samples. (A) SDS-PAGE gel of samples of loaded TK lysate and the flow through fractions. Lane 1: SDS Marker; Lane 2: 2x diluted TK lysate sample; Lane 3-7: wash fractions 1-5; Lane 10: BSA standard reference band with 0.5 mg/ml concentration; (B) Zoomed in images of Lanes 8 and 9 corresponding to eluted TK; Lane 10: BSA standard reference band.


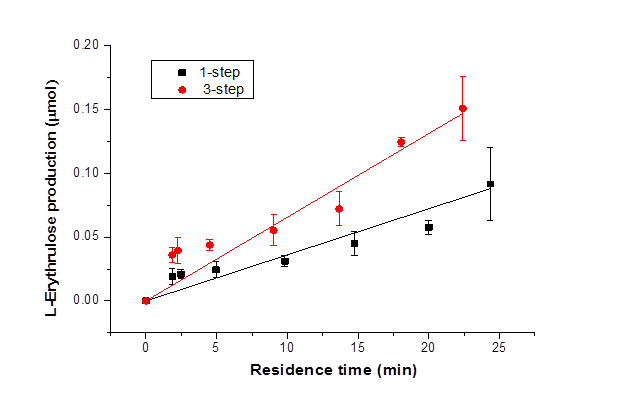


Figure S5. Production of L-erythrulose as function of residence time. The data was obtained from TK-catalysed reaction of 12.5 mM GA and HPA performed at 4◦C in microchannels prepared *via* the 1-step and 3-step immobilization method. Samples were collected in duplicates for each residence time and were analysed for amount of ERY production. The linear range of residence times were taken to calculate the volumetric enzyme activity in corresponding microfluidic devices. Data was fitted with linear regression through the origin and R^2^ values were 0.984 and 0.977 for 1-step and 3-step data, respectively. TK activity values, corresponding to the slope of the fits yielded 0.0036±0.0002 µmol∙min^-1^ for 1-step immobilisation and 0.0066±0.0004 µmol∙min^-1^ for 3-step immobilisation, respectively. TK-catalysed reaction was performed at 12.5 mM glycolaldehyde and hydroxypyruvate substrate concentrations over a range of flow rates from 2.3 to 30 μl∙min^-1^.

**
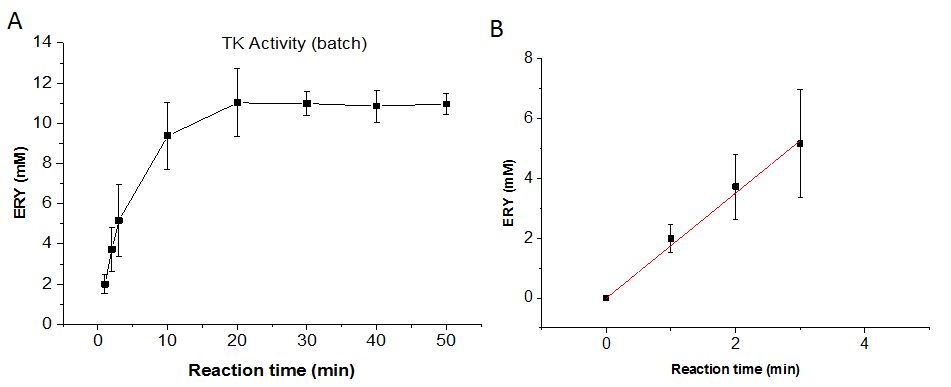
**

Figure S6. Production of L-erythrulose as function of time in batch mode with enzyme in solution. The data was obtained from TK-catalysed reaction of 12.5 mM GA and HPA performed at 4◦C in batch. Samples were collected in duplicates for each time sample and were analysed for amount of ERY produced. Data was fitted with linear regression through the origin and R^2^ value was 0.998. TK activity values, corresponding to the slope of the fitted line yielded 1.76 µmol∙min^-1^ for volumetric enzyme activity that is equivalent to a specific enzyme activity of 3.38±0.08 µmol∙mg^-1^∙min^-1^.

**Figure S7. Productivity of the immobilised poly(methyl methacrylate) (PMMA) microfluidic device (A) and the operational stability of transketolase immoblised via 1-step immobilisation protocol in the device (B).** Reactions were performed using substrate concentrations of 12.5 mM at 4 °C. The productivity is defined as the amount of product generated (per amount of enzyme) divided by the product formed of the starting enzyme activity determined in the same way. A productivity of 100% indicates no denaturation or deactivation of the immobilised enzyme in the microfluidic device. In (B) the average operational productivity of the TK immobilised in the device for the duration of the study was around 70%. Continuous TK reactions were carried out in the PMMA microfluidic device at 10 μl∙min-1 and 5 μl∙min-1, for the productivity (A) and stability (B) studies, respectively.

**Supporting references**

1. Fischer H, Polikarpov I, Craievich AF. Average protein density is a molecular‐weight‐dependent function. Protein Science 2004;13;2825-8.
